# Ionic Liquids as Green and Efficient Desulfurization Media Aiming at Clean Fuel

**DOI:** 10.3390/ijerph21070914

**Published:** 2024-07-12

**Authors:** Peng Wang, Rui Wang, Vitaly Edwardovich Matulis

**Affiliations:** 1 School of Environmental Science and Engineering, Shandong University, No.72 Seaside Road, Qingdao 266237, China; 2 Scientific-Research Institute for Physical Chemical Problems, The Belarusian State University, 220006 Minsk, Belarus

**Keywords:** desulfurization, ionic liquids, fuel, deep eutectic solvent, sulfurous compound, catalytic oxidation

## Abstract

With increasingly stringent emission limits on sulfur and sulfur-containing substances, the reduction and removal of sulfur compounds from fuels has become an urgent task. Emissions of sulfur-containing compounds pose a significant threat to the environment and human health. Ionic liquids (ILs) have attracted much attention in recent years as green solvents and functional materials, and their unique properties make them useful alternatives to conventional desulfurization organic solvents. This paper reviews the advantages and disadvantages of traditional desulfurization technologies such as hydrodesulfurization, oxidative desulfurization, biological desulfurization, adsorptive desulfurization, extractive desulfurization, etc. It focuses on the synthesis of ionic liquids and their applications in oxidative desulfurization, extractive desulfurization, extractive oxidative desulfurization, and catalytic oxidative desulfurization, and it analyzes the problems of ionic liquids that need to be solved urgently in desulfurization, looking forward to the development of sulfuric compounds as a kind of new and emerging green solvent in the field of desulfurization.

## 1. Introduction

Fossil fuels (e.g., gasoline, diesel, and crude oil) have been used since the introduction of machines. Most of these fuels contain sulfur compounds, which have a high capacity to damage production machines. In addition, when they are burned in engines, these fuels release sulfur oxide emissions into the environment [1]. These emissions not only damage facilities and human health, but also contribute to atmospheric pollution, the most common of which are sulfur dioxide (SO_2_) and hydrogen sulfide (H_2_S) [2]. These gasses can form haze and acid rain, causing serious damage to vegetation, soil, and water bodies, disrupting ecological balance, affecting crop growth, and threatening the stability of ecosystems. Secondly, the emission of sulfur compounds can also be harmful to human health. Sulfur compounds such as sulfur dioxide and hydrogen sulfide may trigger respiratory diseases, such as asthma and chronic bronchitis, when they enter the human respiratory system. In addition, prolonged exposure to these gasses may lead to symptoms such as headaches and vomiting, and may even have an effect on the central nervous system [3]. In addition, emissions of sulfur compounds exacerbate the greenhouse effect and accelerate the process of climate change. For another example, common means of transportation will emit exhaust gas containing sulfur compounds, resulting in acid rain [4,5]. At the same time, human beings use a lot of HCFCS in industrial production and life, and the escaping HCFCS are destroying the ozone layer on which we rely [6]. Sulfur compounds react with water vapor in the atmosphere to form sulfuric acid mist, which can lead to changes in the Earth’s radiation balance, exacerbate the trend of global warming, and cause serious impacts on the Earth’s ecosystem and human society [7]. Therefore, controlling the emission of sulfur compounds and minimizing their harm to the environment and human beings has become one of the most important issues for environmental protection and human health.

**Figure 1:**
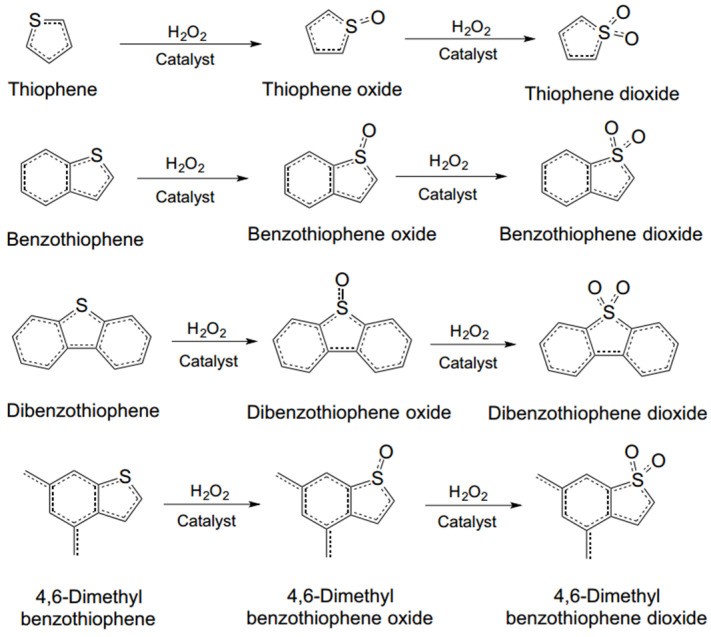
Mechanism of the catalytic reaction of desulfurization [8].

Due to these serious adverse effects, desulfurization must be performed before the use of these fuels. There are many sulfur-containing compounds in the fuel, including thiophene, benzothiophene (BT), dibenzothiophene (DBT), and dimethyldibenzothiophene (DMDBT), as shown in Figure 1 [8]. Various methods have been proposed for this purpose, including biological, ultrasonic, electrochemical, adsorption, extraction, hydrogen, and catalysts under high pressure [9]. However, there are several challenges associated with these methods. These techniques typically involve reacting sulfur-containing compounds with hydrogen at high pressures and temperatures using expensive catalysts [10,11]. Using this method not only lowers the octane rating of gasoline, but also releases toxic hydrogen sulfide (H_2_S) gas [12]. At the same time, the removal of difficult-to-degrade sulfur-containing compounds, such as dibenzothiophene, is particularly challenging [13]. For example, the further reduction of sulfur content using hydrodesulfurization (HDS) would require more stringent conditions than those currently used, which would result in increased capital and operating costs [14]. Furthermore, many have worked to find new alternative methods for the desulfurization of fuels. Researchers have found that ILs show encouraging results. Unlike traditional aqueous solutions or organic solvents, ILs are a new class of low-temperature molten salts that typically consist of organic cations and anions. Their unique structure endows ILs with many excellent properties, such as high stability, melting points close to room temperature, a wide liquid range, strong electrical conductivity, and tunability [15]. Based on these characteristics, functionalized ILs with strong extraction and catalytic ability can be obtained through optimizing the chemical composition and molecular structure. Over the past few decades, ILs have developed rapidly as environmentally friendly alternatives to volatile organic compounds in traditional industrial solvents. These “designer solvents” have an excellent range of properties and their molecular structure can be adapted to chemical process conditions. Therefore, ILs are very suitable as novel media for fuel desulfurization process [16]. In recent years, relevant research has made positive progress in the field of ILs desulfurization, laying a scientific foundation for its application in practice.

However, a comprehensive review of much of the existing literature reveals a relative lack of comprehensive descriptions and evaluations of ILs desulfurization methods. Therefore, this paper describes the various existing desulfurization methods and focuses on the applications and recent advances of ILs in different fuel desulfurization processes. This will help to fill the gap in this research area and provide reference and guidance for future scientific work.

## 2. Overview on Fuels Desulfurization Technologies

### 2.1. Hydrodesulfurization (HDS)

**Figure 2:**
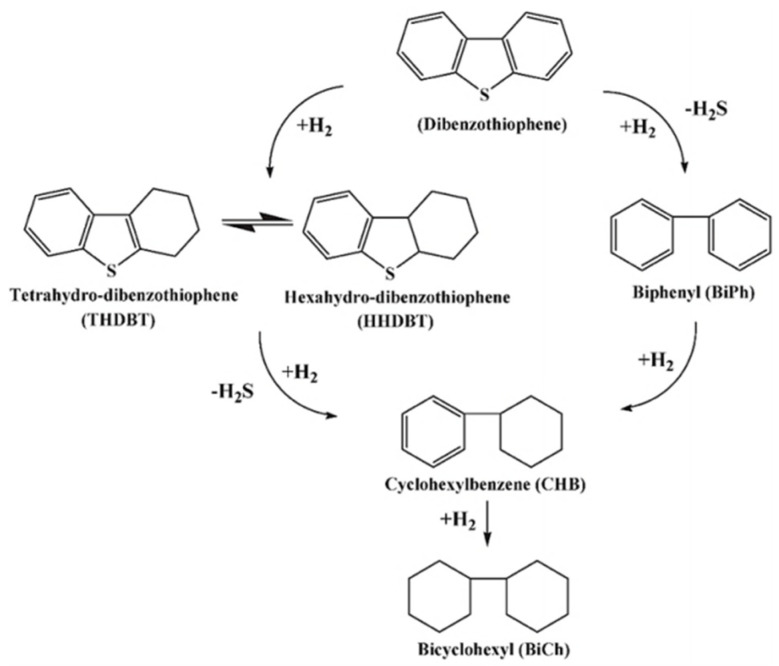
Schematic representation of the hydrogen desulfurization pathway of dibenzothiophene at 300 °C and 102 atm with CoMo/Al_2_O_3_ as catalyst [25].

Hydrodesulfurization (HDS) is the main technology used in refineries. Sulfur, in the form of mercaptan and thiophene, is present in the fuel, which is an undesirable situation. In the HDS process, hydrogen combines with sulfur to form hydrogen sulfide (H_2_S) [17]. Many refineries use HDS technology to reduce SO_2_ emissions [18]. The advantages of this technology are less hydrogen consumption, a short reaction time, high desulfurization efficiency, and a more economical process [19]. The hydrodesulfurization of industrial fuels is mainly based on alumina material catalysts with cobalt, nickel, and molybdenum (e.g., Co–Mo/Al_2_O_3_, Ni–W/Al_2_O_3_ or Ni–Mo/Al_2_O_3_ catalysts) as catalyst carriers. Figure 2 illustrates the HDS process of dibenzothiophene at 300 °C and 102 atm in the presence of CoMo/Al_2_O_3_. Under industrial conditions, the HDS reactivity of sulfur-containing compounds decreased in the following order: Thiophene (T) > Benzothiophene (BT) > Dibenzothiophene (DBT) > 4,6-dimethyldibenzothiophene (4,6-DMDBT) [20]. In order to improve the performance of CoMo- and NiMo types of HDS catalysts, in addition to increasing the content of active metals, other methods can be used to promote the dispersion and distribution of active metals or increase the number of active substances. For example, Kwak et al. reported that the addition of phosphorus can increase the number of active sites, due to the interaction of Mo with P-OH groups and other hydroxyl groups on the catalyst surface, which enhances the dispersibility of Mo [21]. The addition of phosphorus to the CoMo catalyst also increased Bronsted acidity and promoted the migration of methyl substituents in the aromatic ring. Prins et al. found that the addition of fluorine to the Mo/Al_2_O_3_ catalyst did not change the inherent properties of the active site, but changed the dispersion of the active site on the surface [22]. When compared with traditional hydrotreating catalysts, nitrides and carbides have higher catalytic activity, higher selectivity, and lower H_2_ consumption. Transition metal phosphide has similar physical properties to nitrides and carbides, and has attracted much attention in the field of new catalytic materials. In contrast, bulk catalysts with high HDS activity are considered to be novel HDS catalysts due to their high metal content, high active components, and the absence of support. For example, ExxonMobil Research Engineering has developed a bimetallic catalyst consisting of Ni(Co) and W(Mo) through a slurry reaction of nickel carbonate (cobalt) and tungstate (MoO_3_) [23]. During the reaction, the first organic compound (such as ethylenediamine) or the second organic compound (such as citric acid) can be added to the precursor in different steps. The catalysts showed different XRD patterns and hydrogenation activities through thermal decomposition in different atmospheres. Zhang et al. successfully synthesized a series of CoxP/C catalysts with different phases (Co_2_P, CoP) through mofstemplating the phosphorylation of Co/C, and applied them to the HDS of DBT. The prepared CoP/C samples had a high specific surface area (76 m^2^·g^−1^), high cobalt content (16.8 at.%), and an average particle size of 24.2 nm. In the HDS of DBT, the CoP/C catalyst showed better catalytic performance than the Co/C catalyst, with 93.7% DBT conversion, 67.4% stability at 100 h, and 67.4% BP yield at 380 °C under 30 atm [24].

For refiners, the use of highly active catalysts for hydrodesulfurization can avoid equipment modification and is the most cost-effective method. Although the desulfurization effect can be enhanced through increasing the rigor of the hydrodesulfurization (HDS) process conditions, higher pressures may increase the olefin saturation, resulting in a decrease in the octane number of gasoline. Higher temperatures may increase coke production, resulting in catalyst deactivation. Therefore, the development of higher-activity diesel ultradeep hydrodesulfurization catalysts has become an urgent problem to be solved.

### 2.2. Oxidative Desulfurization (ODS)

Oxidative desulfurization (ODS) technology is a deep sulfur removal technology that centers on the oxidation of organic sulfides. The main oxidation methods include H_2_O_2_ oxidation, biological oxidation, photochemical oxidation, and catalytic oxidation [26]. Sulfur in fuel mainly exists in the form of thiophene compounds, which account for about 85% or more of the total sulfur in fuel. The oxidation mechanism of thiophene-like sulfur-containing compounds has been studied. Oxidative desulfurization (ODS) technology can be carried out at ambient temperatures and pressures without hydrogen and with less equipment investment [27]. For dibenzothiophene compounds, which are difficult to be removed using traditional hydrodesulfurization technology, ODS technology shows high desulfurization efficiency and can meet the demand of deep desulfurization, so it is known as the green desulfurization process of the 21st century with a broad prospect.

**Figure 3:**
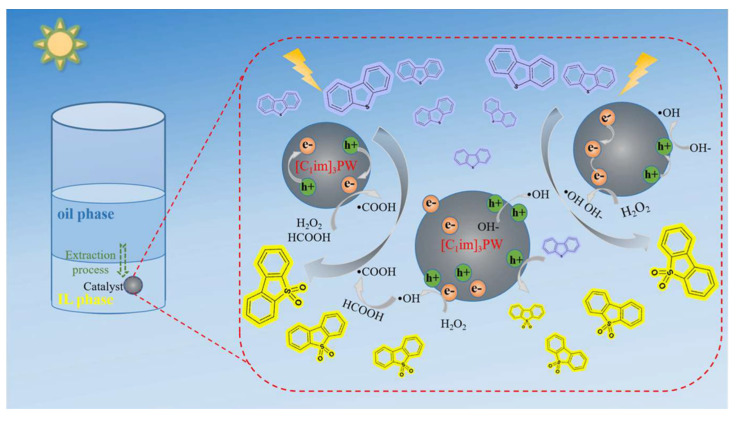
Reaction process and mechanism of EPODS system [30].

Otsuki, a well-known scholar, found through research that hydrogen peroxide has a fourth-level oxidation activity that is the opposite to hydrodesulfurization, so the sulfur compounds that cannot be removed via hydrodesulfurization can be effectively removed when hydrogen peroxide is used for desulfurization [28]. The hydrogen peroxide system is applied more frequently during deep desulfurization, while combining it with organic acids improves sulfur removal. Photocatalytic oxidation utilizes external light irradiation, and, when the energy of the incident photon is greater than the band gap energy of the material, it excites valence band electrons to the conduction band, generating electron–hole pairs with strong reactivity. These electron–hole pairs migrate to the surface of the particle, accelerating the REDOX reaction. Photocatalytic oxidation has the advantages of good selectivity, a wide application range, and can be carried out under ambient temperatures and pressures [29]. Liu et al. prepared a phosphotungstic acid-based imidazolium ILs, [C_1_imCH_2_CH_2_COOH]_3_PW_12_O_40_ ([C_1_im]_3_PW), and used it as a photocatalyst in an extraction-coupled photocatalytic oxidative desulfurization (EPODS) system consisting of [OMIM]PF_6_, H_2_O_2,_ and formic acid [30]. The researchers studied the structure and composition characteristics of [C_1_im]_3_PW, and found that it has excellent photocatalytic properties under visible light. The desulfurization rate of dibenzothiophene could reach 99.8% in 30 min at room temperature and atmospheric pressure. At the same time, they also studied the EPODS system’s reusability, anti-interference, desulfurization reaction process, and possible mechanism. Figure 3 shows possible EPODS system response processes and mechanisms.

### 2.3. Biodesulfurization (BDS)

**Figure 4:**
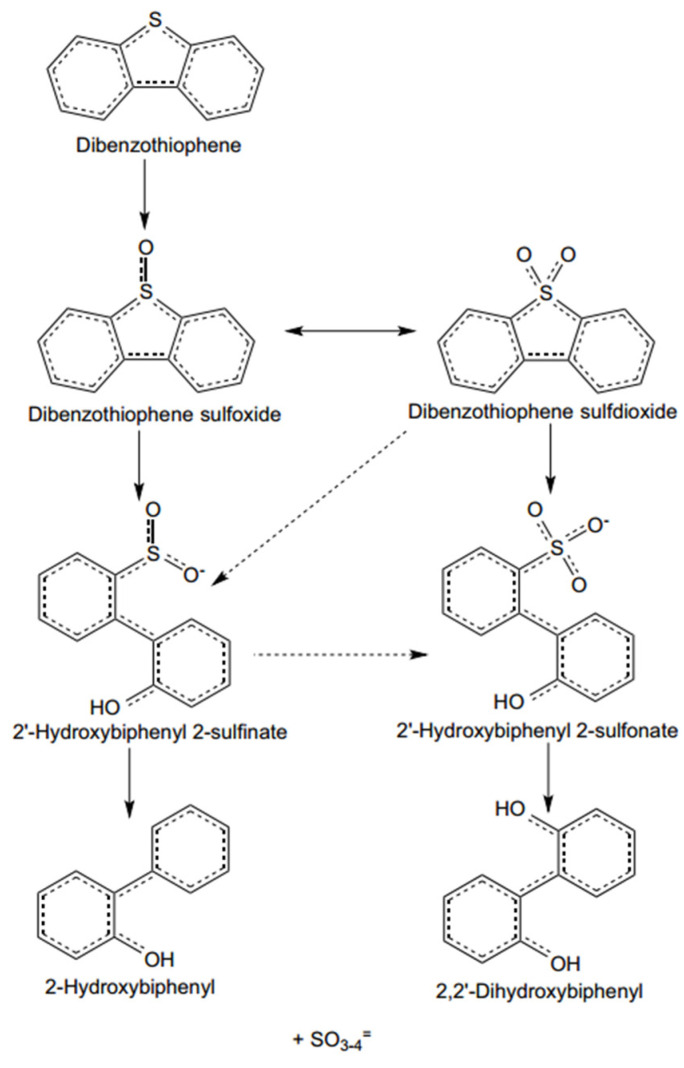
Schematic diagram of dibenzothiophene biodesulfurization mechanism [8].

Biodesulfurization (BDS) is a new technology designed to isolate bacteria with sulfur-baiting properties from industrial sludge or waste [5]. These bacteria grow in a sulfur-free environment and are introduced into fuel oil. These strains selectively utilize the sulfur content to convert dibenzothiophene (DBT), benzothiophene (BT), and thiophene analogs to 2-hydroxybiphenyl (HBP) and other less harmful compounds [31]. The mechanism of dibenzothiophene biodesulfurization is shown in Figure 4. The effectiveness of desulfurization depends on the activity of the strain. The treatment of fuels with ultra-low sulfur content is possible with this technology. Bacteria that reduce sulfur by degrading sulfides or converting sulfur into separable species are primarily derived from petroleum, oil fields, soils, and waste oil, sewage, or water bodies.

While other alternatives are being developed to desulfurize various refinery products, BDS will be a major breakthrough in the process [31]. BDS is expected to be a low-cost alternative to HDS, resulting in lower capital and operating costs [32]. Given the growing interest in reducing greenhouse gas emissions in light of the requirements of the Kyoto Protocol, it has been calculated that the use of BDS rather than HDS can reduce CO_2_ emissions and energy demand [33]. Therefore, BDS is attracting attention as a promising alternative to conventional HDS in refineries.

Therefore, BDS is attracting attention as a promising alternative to conventional HDS in refineries. Since site-resistant alkyl DBT is less reactive in HDS but is the preferred substrate for BDS, BDS can be used as a complement to the hydrotreating process. Therefore, BDS should be considered as a complementary technology for the removal of recalcitrant molecules from HDS-treated oils rather than an alternative technology. In order to achieve very low sulfur levels in diesel fuels, it has been suggested that a combination of BDS and conventional HDS technology is effective [31].

“Pantoea agglomerans D23W3” is a bacterium collected from an oil refinery and has been used for diesel desulphurization, where it showed 22% desulphurization on lignite [32]. In addition, Pseudomonas aeruginosa obtained from an oil field in China has been immobilized on calcium alginate microspheres. These immobilized cells can be recycled up to 15 times for biological desulfurization, with a total desulfurization time of up to 450 h. These immobilized cells significantly degrade both thiophene and DBT, achieving desulfurization efficiencies of up to 40% and 25%, respectively [33].

### 2.4. Adsorption (ADS)

Another option for removing sulfur from fuel is through an adsorption process. In this process, sulfur compounds are selectively adsorbed by the adsorbent without any chemical reaction. The surface area of the adsorbent can be increased by placing it on a porous and non-reactive substrate [34]. Advantages of adsorption desulfurization include mild operating temperatures [5], no hydrogen involvement, and low sulfur levels can be achieved [35]. This method reduces capital and operating costs, and produces a high-value by-product. However, adsorption has some drawbacks, such as slow reaction rates [36], poor selectivity of sulfur compounds, and a low adsorption capacity of many adsorbents [10].

The ideal adsorbent should have the ability to adsorb sulfur-containing compounds quickly and have a high adsorption capacity. In addition, it should be easy to regenerate. The selective adsorption of aromatic hydrocarbon sulfur compounds by adsorbents without the adsorption of other aromatic hydrocarbons in the fuel is also an important challenge. Although adsorption can be efficient, the regeneration process of the adsorbent is relatively limited because the solvent washing or calcination of the adsorbent is usually required. Many adsorbents have been reported to have a low adsorption capacity, and thus large and multiple adsorption beds may be required to minimize disruptions in the replacement and maintenance process [10]. Therefore, much work has been devoted to the development of low-cost, high-surface area adsorbent materials. Guo et al. introduced Fe, Co, and Zn metal ions into the MCM-41 molecular sieve skeleton via a hydrothermal method to maintain the complete MCM-41 molecular sieve configuration. The ADS properties of zeolite were improved by the introduction of metal ions. Fe/MCM-41 molecular siolite has the best desulfurization performance, with an equilibrium adsorption capacity of 14.02 mg/g and a desulphurization rate of 90% [37].

### 2.5. Extraction (EDS)

**Figure 5:**
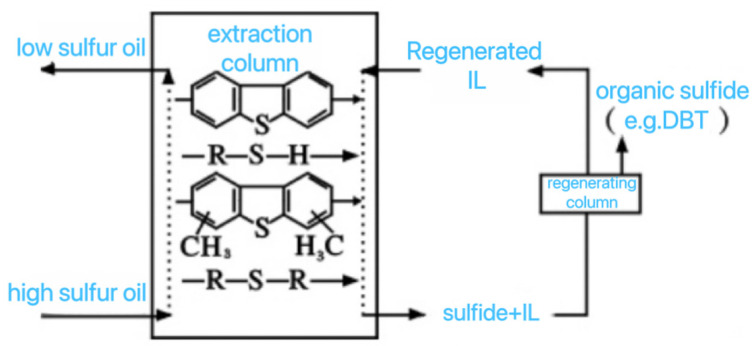
IL Schematic diagram of the extractive desulfurization process [41].

The ILs extraction desulfurization process is shown in Figure 5. The most commonly used gasoline desulfurization extraction solvents in research include alcohols, aldehydes, amines, sulfones, and prides, which are usually mixed with water or new ILs. Traditional extractants include solvents such as pyrrolidone, acetonitrile (AcN), dimethylformamide (DMF), and dimethyl sulfoxide (DMSO) [38]. However, traditional extraction solvents have some problems, such as high volatility, high toxicity, and environmental risks. In contrast, EDS (electron impact dissociation) technology has advantages and disadvantages. Its advantages are that the reaction conditions are mild and no auxiliary substances such as hydrogen or catalysts are required. In addition, EDS can selectively extract sulfur compounds from fuel oil without reacting with other desired hydrocarbons in the fuel oil [13]. Selectivity is important when selecting extractants because of the similar polarity of aromatic sulfur-containing compounds and aromatic sulfur-free hydrocarbons in fuels [36]. In future research, it is necessary to design a functional ILs extraction desulfurization system with a low cost, high selectivity, and good stability, so as to significantly improve the desulfurization selectivity and desulfurization efficiency of real oil products [39].

For example, He et al. designed a novel porous ILs (M-BN-PIL) using butyl pyridine tetrachloroferrate ([BPy][FeCl_4_]) as an organic guest and microporous boron nitride (m-BN) as a porous skeleton [40]. The formation of porous ionic liquids (PIL) promotes electron transport between [BPy][FeCl_4_] and m-BN, thereby enhancing pyridine cyclocation ([BPy]^+^) and promoting m-BN dispersion, which facilitates the exposure of extraction sites. Further characterization verified that m-BN-PIL remained stable during extraction. Using m-BN-PIL as an extractor, the desulfurization properties of dibenzothiophene (DBT) and 4-methyldibenzothiophene (4-MDBT) reached 59.2% and 61.8%, respectively.

## 3. Ionic Liquids and Their Application in the Desulfurization Process

### 3.1. Ionic Liquids

ILs are liquid substances composed of ions with melting points below or near room temperature. These are usually composed of organic cations and inorganic anions and are electrically neutral [15]. The inter-ionic interaction forces are mainly realized through Coulombic interactions, and the magnitude of the interaction is related to the number of charges and radii of the anions and cations. When the ion radius is larger, the inter-ionic force becomes smaller accordingly [42]. Since the internal force of ILs is mainly dominated by ionic bonding, it has some physicochemical properties that are completely different from those of traditional molecular solvents, such as a very low vapor pressure, good thermal stability, and high solubility. Many types of ILs have been identified and can be categorized according to the type of anion and cation. Cations include imidazole ions, alcoholamine ions, guanidine ions, and quaternary amine ions, and anions include boric acid ions, carboxylic acid ions, and amino acid ions [43]. Due to their special form of ionic structure, ILs play an important role in the fields of biomass conversion, catalytic reaction, organic synthesis, and gas absorption [44].

ILs have very high thermal stability and strong electrical conductivity, and present a liquid state at room temperature. They are very weak in a certain temperature range of volatility and can avoid solvent losses caused by solvent volatilization, thus, concerning cost savings and environmental protection, has a greater advantage. Secondly, the anions and cations in ILs can be easily replaced or modified, so ILs have unique and variable properties. Through different modifications to the anion or cation, such as adding some specific functional groups or adjusting the combination of anions and cations, ILs with different properties can be obtained. Meanwhile, ILs have catalytic effects on certain reactions. Therefore, choosing the appropriate IL as the reaction system can not only be used as the reaction solvent, but can also have a certain catalytic effect, which can save a lot of costs [45].

Due to the structural variability of chemical substances, researchers and the industry must consider not only their technological advantages, but also their (eco)toxicological risks [46]. While ILs may help to reduce the risk of air pollution, their potential toxicity and non-biodegradability cannot be ignored [47]. For example, the release of ILs into the aquatic environment may cause serious water pollution. The acute and chronic toxicity of imidazolium-based cationic ILs to aquatic organisms was first studied by Bernot et al. [48]. Through their studies, they found that the toxicity of imidazolium-based ILs (containing various anions such as Cl^−^, Br^−^, [PF_6_]^−^, and [BF_4_]^−^) was similar to that of solvents commonly used in the chemical industry, such as ammonia and phenol. They also noted that if these salts were to leak out, they could be more damaging to aquatic ecosystems than traditional volatile organic solvents. All ILs showed a higher toxicity when compared to the data for inorganic sodium salts containing the same anion. The fact that the ILs commonly used to date are toxic in nature has been demonstrated by a variety of toxicological data collections for a wide range of organisms. As toxicologists and synthetic chemists work closely together, more truly green and efficient ILs will emerge [49].

According to the structural characteristics of ILs, they can be divided into two categories as follows: conventional ILs and functional ILs. Conventional ILs are mainly used in the study of removing acidic gasses, in which imidazole ILs dominate [50]. Conventional ILs mainly remove acidic gasses via physical absorption, so their absorption capacity is poor, while functional ILs introduce functional groups into the structure of conventional ILs, so that they have special functions [51]. Most of the functional ILs used to absorb acidic gasses have alkaline groups which can chemically react with acidic gasses, and thus have good absorption performance. Designing and synthesizing ILs with clear targets and special functions has become one of the hot spots in today’s ILs research.

### 3.2. Synthesis of Ionic Liquids

#### 3.2.1. One-Step Synthesis of Ionic Liquids

Usually, in most cases, only one step is required to synthesize an IL. The one-step synthesis method consists of the one-step generation of the target ILs from a nucleophilic reagent—a tertiary amine (including pyridine, imidazole, etc.)—by a nucleophilic addition reaction with a halogenated alkane or ester (carboxylic acid ester, phosphate ester, and sulfate ester) or by using the basicity of the amine to react with an acid in a neutralization reaction. Examples include nitroethylamine ILs and tetrafluoroborate ILs [52]. However, many counteractions are generated as amphoteric ions and further react with the desired anion to form highly viscous products. For example, Wu et al. synthesized imidazolium-type quaternary ILs in a one-step process [53]. Zhang et al. synthesized tetramethylguanidine 1,1,3,3 lactate (TMGL) and 1-butyl-3methylimidazolium hexafluorophosphate ([BMIM]PF_6_) in a one-step process and tested their thermodynamic properties [54].

#### 3.2.2. Two-Step Synthesis of Ionic Liquids

**Figure 6:**
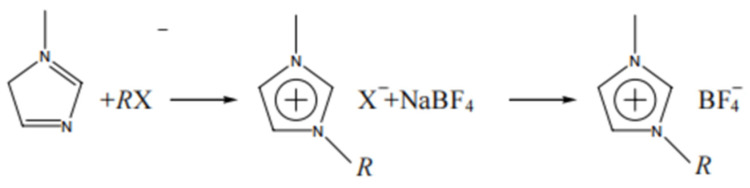
The illustration of the preparation of [BMIM]^+^[BF_4_]^−^ [55].

When the target ILs cannot be obtained through one-step synthesis, the two-step synthesis method can be used. The route of the two-step synthesis method is shown below. The first step is to synthesize quaternary ammonium halide via the reaction of tertiary amine and halogenated hydrocarbon. The second step is to replace the halide ions with the anions of the target ILs and perform electrolysis or a complexation reaction. For example, Li et al. synthesized the illustration of the preparation of [BMIM]^+^[BF_4_]^−^ using N-methylimidazole and brooksubstituted n-butane as starting materials in a two-step process [55]. The synthesis process is shown in Figure 6. Wang et al. synthesized a novel imidazole ILs salt, 1-allyl-3-methylimidazole hydrogencarbonate of sulfuric acid ([EMIM]HSO_4_), using a two-step synthetic method and determined its chemical structure and properties [56].

#### 3.2.3. Microwave Synthesis of Ionic Liquids

**Figure 7:**
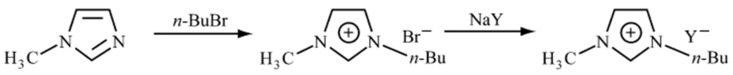
Two-step synthesis of rosin ethyl ester [59].

The microwave method utilizes a rapidly changing electromagnetic field to cause polar molecules to constantly change direction, thus generating frictional heating, which is a method of body phase heating. This heating method is characterized by a fast heating speed, which can significantly improve the reaction rate, with some reactions even being completed in a few minutes. In addition, the microwave method can also improve the yield and purity of the product. One of the first methods to synthesize imidazolium-based ILs in a household microwave oven was reported by Varma et al. [57]. They mixed [BMIM]Cl with NH_4_BF_4_ at a substance-to-mass ratio of 1:1.05, and reacted the mixture for a few minutes under a microwave fire of 360 W. [BMIM]BF_4_ was obtained in 81% to 91% yields. Hsiao et al. successfully synthesized [BMIM]PF_6_ ILs using the microwave method, which shortened the synthesis time of traditional ILs and increased the yield [58]. The reaction time was shortened from the original 24 to 48 h to 1 h. The optimum yield of [BMIM]PF_6_ ILs was about 49.5%. Subsequently, many people improved the synthesis method on this basis. For example, Xu et al. synthesized rosin ethyl ester via the direct esterification of rosin and ethanol under microwave radiation using 1-n-butyl-3-methylimidazole p-toluenesulfonate ILs ([BMIM]Pt_SA_) as the solvent and catalyst, and determined the optimal reaction conditions as follows: the reaction temperature is 100 °C, the reaction time is 1 h, the mass ratio of [BMIM]Pt_SA_ and rosin is 4:1, and the rate of esterification reached 95.3% in this optimal condition [59]. The synthesis method is shown in Figure 7. The esterification rate reached 95.5%. It was found that the ILs is easy to separate from the reaction product and can be reused.

#### 3.2.4. Ultrasonic Synthesis of Ionic Liquids

**Figure 8:**
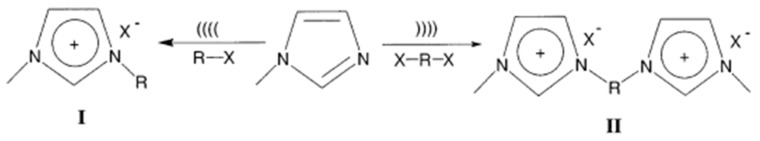
Ultrasound-promoted preparation of 1-Alkyl-3-Methylimidazolium Halides [61].

Ultrasonic methods can use ultrasonic cavitation to form a local high-temperature and high-pressure mixed environment inside the liquid, and the vibration stirring effect of ultrasonic can significantly improve the reaction rate, especially for heterogeneous chemical reactions. Hsiao et al. modified traditional ultrasonic synthesis methods to increase the yield of ILs [60]. In the first phase of the synthesis reaction, they placed 0.3 mol of 1-methylimidazole and 0.3 mol of 1-chlorobutane into a heating reflux device, controlled at a temperature in the range of 50–90 °C, and performed ultrasonic synthesis for 60–30 min. After the reaction, the synthetic product is cooled to room temperature and mixed with a small amount of ethyl acetate. The cloudy ethyl acetate is then removed from the upper layer by repeating the stratified extraction process 5–8 times until the upper-layer liquid is completely clarified. Finally, a vacuum dryer is used to remove excess ethyl acetate. In the second stage of the synthesis reaction, they dissolved 0.3 mol of KPF6 in acetone and added the synthesized product in the first stage. At the end of the reaction, most of the salt is removed through suction filtration, then a vacuum dryer is used to remove any excess acetone. The results of the ultrasonic-assisted synthesis of ILs show that the optimal ultrasonic output power of [Bmim]Cl is 100%, the reaction time is 120 min, and the optimal yield is 61%. When compared with the traditional 48–72 h synthesis method, ultrasonic-assisted synthesis shows a more efficient effect. In the second stage, the ultrasonic-assisted synthesis of [Bmim]PF_6_ also showed significant advantages, as the reaction time was reduced from the traditional 24 to 48 h to just 1 h, and the yield reached 49% (at 80 °C). These results indicate that the ultrasonic-assisted synthesis of ILs is not only faster, but also more energy-efficient. Subsequently, more researchers proposed more efficient synthesis methods. Namboodiri et al. synthesized brominated 1,3-dialkylimidazole ILs using ultrasonic waves as an energy source under solvent-free conditions in a closed system, and the yields were all greater than 90% [61]. The reaction process is shown in Figure 8. Two methods showed that the ultrasound-assisted method significantly improved the reaction efficiency. The ultrasound-assisted method has mild conditions, a high efficiency, and has broad application prospects in IL synthesis.

### 3.3. Application of Ionic Liquids in Fuel Desulfurization Processes

#### 3.3.1. Ionic Liquid Oxidative Desulfurization

Oxidative desulfurization (ODS) is a non-hydrodesulfurization method that can be performed at low temperatures (~50 °C) and atmospheric pressures without the use of hydrogen, and is one of the most beneficial technologies [62]. The ODS process involves two major steps as follows: first, sulfur-containing compounds are oxidized by oxidizing agents (e.g., H_2_O_2_ [63], O_2_ [64], O_3_ [65], C_4_HO_2_, peroxyacid, and NO_2_) to their sulfone or sulfoxide derivatives. Secondly, the resulting more polar sulfur oxide compounds are removed from the reaction mixture via adsorption or liquid–liquid extraction. In this new method, IL replaces the traditional organic solvent as the extractant. In this process, sulfur-containing compounds enter the ILs phase and are converted to sulfonic acid compounds after oxidation. Due to its high polarity, sulfone is easily removed.

Chu’s study showed that when the ratio of ionic liquid-to-oil fuel specimen was 1:2, the desulfurization rate of [C_8_MIM] was 18% and [BF_4_] was 5% [66]. This result indicates that the longer the structural length of the imidazole, the better the desulfurization effect. For thiophene sulfide, due to the difference in its spatial structure, direct extraction can not be effectively removed, so the use of the oxidation method—first, thiophene sulfide oxidation into sulfone, then dissolved extraction, and finally using physical means to separate the sulfur element so as to obtain sulfur-free petroleum fuel—is preferred. Nie’s research shows that adding iron ions to the oxidation reaction can effectively improve the degree of desulfurization, and the reaction rate can even reach equilibrium within one minute at 25 °C air pressure [67]. David’s research shows that, under the control of the reaction temperature of 30 °C and the air pressure of 0.99 atm, and with the ratio of the ionic liquid-to-blended oil being 1:5, the effect of desulfurization can reach 95% in 20 min. Zhang et al. synthesized and characterized three amphiphilic phosphomolybdates, namely [C_4_MIM]_3_PMo_4_O_24_, [C_8_MIM]_3_PMo_4_O_24_, and [C_16_MIM]_3_PMo_4_O_24_ [68]. These catalysts used H_2_O_2_ as an oxidant and ILs [C_4_MIM]BF_4_ as an extractant for fuel extraction and catalytic oxidation desulfurization. The results showed that [C_16_MIM]_3_PMo_4_O_24_ had the highest catalytic activity and was able to reduce the sulfur content to 7.5 ppm. In contrast, the performance of the desulfurization system without H_2_O_2_ or ILs was poor. The reaction conditions, such as the reaction time, temperature, the amount of H_2_O_2_ and catalysts, and different types of sulfur compounds, were optimized in detail. After the reaction, the catalyst and ILs can be recycled up to eight times, but the desulfurization efficiency is slightly reduced.

#### 3.3.2. Ionic Liquid Extraction Desulfurization

ILs extraction desulfurization is a method of extracting sulfur-containing compounds from fuel oil into an ILs and oxidizing them to the corresponding sulfone or sulfoxide in the presence of a catalyst and oxidizer [69].

**Figure 9:**
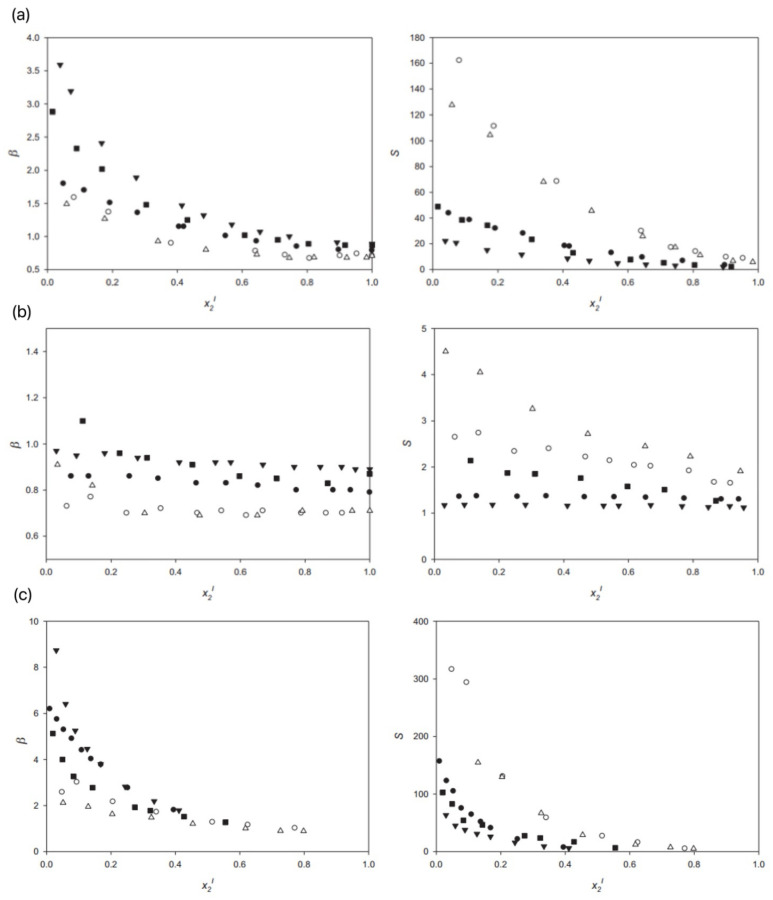
(**a**) β (left) and S (right) values for the ternary system {IL + thiophene + n-hexane} at 298.15 K and atmospheric pressure. The compared ILs are as follows: [C2MIM][NTf2] (●), [C2MIM][EtSO4] (⚪), [C6 2,4mmpy][NTf2] (▼), [C2MIM][OAc] (△), and [C2MIM][DEP] (■); (**b**) β (left) and S (right) values for the ternary system {IL + thiophene + toluene} at 298.15 K and atmospheric pressure. The compared ILs are as follows: [C2MIM][NTf2] (●), [C2MIM][EtSO4] (⚪), [C6 2,4mmpy][NTf2] (▼), [C2MIM][OAc] (△), and [C2MIM][DEP] (■); (**c**) β (left) and S (right) values for the ternary system {IL + pyridine + n-hexane} at 298.15 K and atmospheric pressure. The compared ILs are as follows: [C2MIM][NTf2] (●), [C2MIM][EtSO4] (⚪), [C6 2,4mmpy][NTf2] (▼), [C2MIM][OAc] (△), and [C2MIM][DEP] (■) [71].

Hu et al. used different types of ILs to extract sulfur-containing organic compounds to remove sulfur from simulated oils [70]. They observed the effect of the ILs type and dosage on the desulfurization effect. The results showed that the ILs extraction could reach equilibrium within 10 min. As the volume ratio of ionic liquid-to-oil phase increased, the desulfurization effect improved significantly. Cations and ions had a great influence on the desulfurization effect, and the specific ILs 1-xylene-3-methylimidazolium fluoride hexaphosphate ([BMIM]PF_6_) had the best desulfurization effect, while the hydrophilic ILs 1-xylene tetrakis-3-methylimidazolium tetrafluoroborate ([BMIM]BF_n_) had a weak desulfurization effect. When the volume ratio of ILs-to-oil was 1, [BMIM]PF_6_ and [BMIM]BF_4_ extracted purified material from diesel fuel, reducing the mass fraction of sulfur from 5.43% to 2.91% and 4.10%, respectively. Borja and his team showed that the ILs 1-ethyl-3-methylimidazolium bis(trifluoromethylsulfo-nyl)imide had better selectivity for this application and could be a good alternative [71]. To reduce the sulfur content in the anion, they investigated the desulfurization ability of two other 1-ethyl-3-methylimidazole ILs, namely acetate and diethyl phosphate. At 25 °C and an atmospheric pressure, they obtained liquid–liquid equilibrium data for the ternary systems of ILs + thiophene + toluene, ILs + thiophene + n-hexane, and ILs + pyridine + n-hexane, and evaluated the effect of ILs anions on the extraction process via the solute distribution ratio and selectivity. Figure 9 shows the solute distribution ratio and S value of the ternary system. In a mixed solution of n-pentane and toluene, Zeng et al. used imidazole-based ILs as desulfurizers and added a small amount of thiophenazole to construct an oil simulation system [72]. Through orthogonal experiments, the effects of the temperature, time, oil ratio, and IL carbon number on the desulfurization efficiency of single-stage extraction were studied. The optimal desulfurization conditions were obtained as follows: temperature about 40 °C, reaction time about 50 min, agent-to-oil ratio 1:1, and side chain carbon number 10. This study provides an important foundation for the desulfurization process based on ionic fuel oil.

#### 3.3.3. Ionic Liquid Extraction Oxidative Desulfurization

Extractive oxidative desulfurization (EODS) is a process for the treatment of sulfur-containing oxides and is considered one of the most effective processes. Research results show that EODS is superior to edds [73]. At present, the extraction method is widely used in the world to treat sulfur oxides, in which H_2_O_2_ and the IL participate in the EODS process as an oxidant and extractant, respectively [74]. In this process, sulfur compounds are oxidized and converted to the corresponding sulfone compounds. With the help of organic solvents, these products can be selectively extracted and separated. ILs play two roles in this process as follow: first, it serves as an extraction medium for sulfur-containing compounds, which can be preferentially extracted into ILs due to their greater solubility in ILs than in fuel oils; and second, the ILs also provide catalytic oxidation conditions for the conversion and oxidation of sulfur-containing compounds [69]. The commonly used ILs cations are imidazoles, while the anions are acid esters, fluorophosphoric acid, Lewis acid, fluoroboric acid, etc., with a wide selection range [41]. Commonly used oxidizing agents include O_2_, O_3_, H_2_O_2_, nitrogen oxides, and tert-butyl hydroperoxide [69].

**Figure 10:**
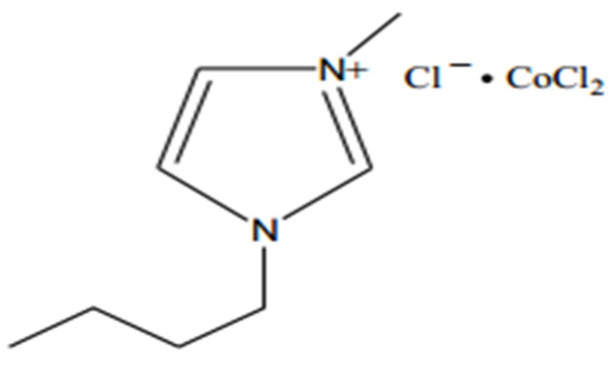
Chemical structure of 1-n-butyl-3-methylimidazolium cobalt chloride [79].

Lo et al. used [BMIM]BF_4_ and [BMIM]PF_6_ as extractants for the treatment of simulated light crude oil, consisting of tetradecane and dibenzothiophene (DBT) with a mixture of hydroxide and acetic acid (HAc) as oxidizing agents [75]. The results of the study showed that the best results were obtained using [BMIM]PF_6_ as the extractant. After 6 h of continuous extraction, the DBT content in the simulated light crude oil decreased from 758 × 10^−6^ to 7.8 × 10^−6^ with a desulfurization rate of 99%. Yao et al. showed that, when compared with ILs with histidine, glycine, and tryptophan as anions, the H_2_O_2_ system with aspartic acid as an anion had the best desulfurization effect [76]. Under the conditions of the optimal reaction temperature, the H_2_O_2_-to-simulated crude oil volume ratio, and reaction time, the desulfurization rate could reach 96.5%. After seven cycles and repeated uses, the desulfurization rate of [C__8_MIM]Asp ILs can still be maintained at 93.7%. Cui et al. used laboratory-synthesized Zn[C_6_H_11_NO]_3_Cl_2_, N-methylimidazole bromide, Zn [CO(NH_3_)_2_]_3_Cl_2_, 1-butyl-3-methylimidazole tetrafluoroborate, and phosphate ILs as a desulfurization extractant, and, at the same time, using the H_2_O_2_–glacial acetic acid system as an oxidizing agent through the “one-pot method”. The desulfurization of straight-run diesel was carried out using the “one-pot method” [77]. The researchers studied the effects of the type and amount of ILs, the amount of H_2_O_2_ and glacial acetic acid, the reaction time, and the oxidation temperature on the desulfurization rate. The results showed that phosphate-based ILs were more effective in the desulfurization of straight-run diesel fuel, and the sulfur mass fraction in diesel fuel was reduced from 1425 μg/g to 676 μg/g, with a desulfurization rate of 52.6% under the conditions of a 20% volume fraction of IL, an 8% volume fraction of H_2_O_2_, a 4% volume fraction of glacial acetic acid, a reaction time of 20 min, and a reaction temperature of 80 °C. After five cycles, the desulfurization efficiency could still be maintained above 40%. Jiang et al. synthesized a series of acidic ILs, namely [C_2_OHmpip]FeCl_4_, [C_4_mpip]FeCl_4_, [C_8_mpip]FeCl_4,_ and [C_12_mpip]FeCl_4_, and investigated the extractive oxidative activity of DBT in simulated crude oil [78]. The results showed that [C_4_mpip]FeCl_4_ had the highest desulfurization rate due to the effect of cation spatial site resistance. When the dosage of ILs was 0.078 mol, the oxygen-to-sulfur ratio was 3.5, the reaction temperature was 60 °C, and the reaction time was 60 min, the DBT removal rate reached 97.1%. Using KHSO_5_ as an oxidant, Xu and his team prepared the ILs [C_4_MIMCl]CoCl_2_ containing acidity, and applied it to simulate the oxidative desulfurization process of oil, as shown in Figure 10 [79]. The results show that the sulfate radicals generated by the reaction of Co^2+^ with KHSO_5_ are strongly oxidizing and help to promote the desulfurization reaction. However, when the amount of ILs is too much, the additional Co^2+^ acts as a radical scavenger, thus hindering the desulfurization process. The experimental results showed that the best desulfurization effect was achieved when the mass of ILs was 0.2 g. The removal rate of diphenyl sulfide reached 97.7%, and the stability was still maintained at over 96% after six consecutive cycles. Mohammed et al. carried out the oxidative extractive desulfurization of diesel fuel using H_2_O_2_ as an oxidant [80]. They determined the thermodynamic parameters of the oxidation reaction and employed the extraction desulfurization process under the conditions of acetonitrile as an organic solvent and a solvent-to-feedstock volume ratio of 1:1, and successfully achieved a desulfurization efficiency of 84.7%. The ILs prepared in the study can be reused at least six times, and its desulfurization performance and chemical structure basically remain stable, showing good reuse characteristics.

#### 3.3.4. Ionic Liquid Catalytic Oxidative Desulfurization

ILs have a dual function as both solvents and catalysts, with many advantages, such as being environmentally friendly, having a controllable structure and acidity, the easy separation of products, and recycling capability, etc. ILs can replace a variety of desulfurization catalysts to reduce the sulfur content of fuel [35]. Catalytic oxidative desulfurization (COD) is an ideal oxidative desulfurization (OD) method that mainly utilizes the extraction and catalytic effects of ILs to enhance the interaction between oxidants and sulfur-containing compounds, thus significantly improving the efficiency of desulfurization [81]. At present, the ILs applied to catalytic oxidative desulfurization are mostly acidic due to their solubility and the catalytic effect of acid roots on the oxidation process. Among them, molybdenum-containing polymetallic oxonate ILs are common, and their structures usually contain quaternary ammonium cations, which can form π-π complexes with sulfur-containing compounds, such as thiophene, during the oxidation process and extract them into the ILs. The anions, on the other hand, can combine with oxidants such as H_2_O_2_ to release oxygen ions, and catalyze the reaction between the two to achieve the desired desulfurization effect [82].

**Figure 11:**
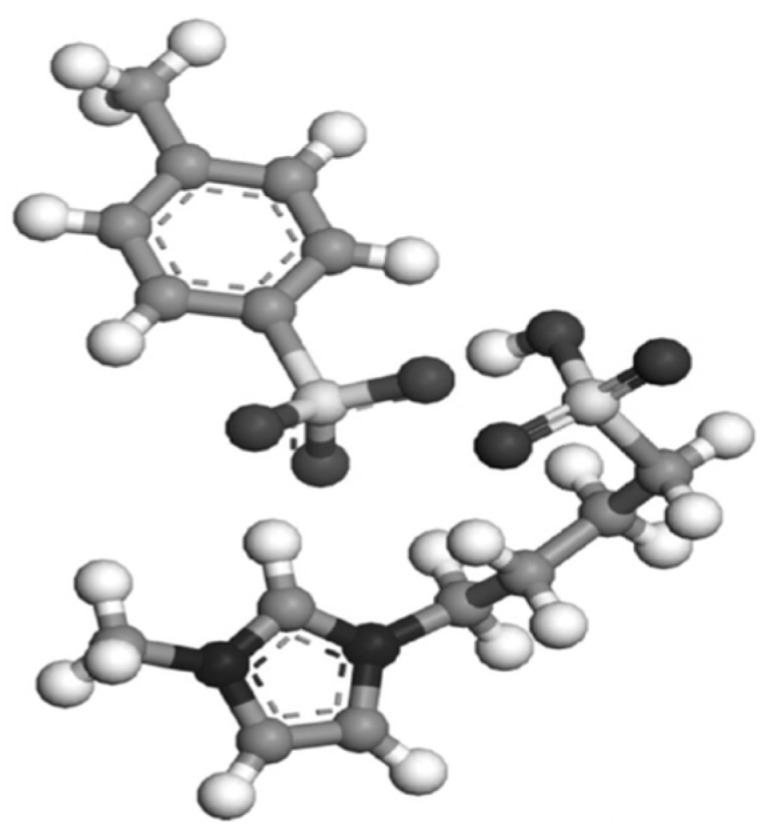
The structure of the ionic liquid [(CH_2_)_4_SO_3_HMIM][Tos] [84].

Zhang et al. synthesized three amphiphilic perphosphates, namely [C_4_MIM]_3_PMo_4_O_24_, [C_8_MIM]_3_PMo_4_O_24_, and [C_16_MIM]_3_PMo_4_O_24_, respectively [68]. The results of the study showed that, among them, [C_16_MIM]_3_PMo_4_O_24_ had the highest catalytic activity, which could reduce the sulfur content to 7.5 ppm compared to the performance of the desulfurization system without H_2_O_2_ or ILs. After the reaction, the catalyst and ILs could be recycled eight times, with a slight decrease in the desulfurization efficiency. On the other hand, An et al. carried out a simulated oil oxidative desulfurization reaction using [HMIM]_3_PMo_12_O_40_ as the catalyst and 1-methylimidazolium tetrafluoroborate ([HMIM]BF_4_) as the solvent [83]. The experimental results showed that the Keggin-type phosphomolybdenum heteropolyacid IL [HMIM]_3_PMo_12_O_40_ could increase the oxidative desulfurization rate of dibenzothiophene up to 90% at 50 °C at a molar ratio of hydrogen peroxide-to-sulfur of 4. The oxidative desulfurization rate at a molar ratio of hydrogen peroxide-to-sulfur of 10 could reach 100%, which was much higher than that of [HMIM]BF_4_ alone. The catalyst was easy to separate from the oil, and the desulfurization rate did not decrease significantly after four consecutive cycles. A deep oxidative desulfurization process for diesel pre-hydrogenation with Na_2_WO_4_ and ILs as catalysts was investigated in a study by Liu et al. [84], as shown in Figure 11. The catalyst showed good oxidative catalytic activity under mild conditions at a lower dosage of ILs. After the oxidative desulfurization process, the sulfur content in the actual diesel fuel was reduced from the initial 200 ppm to 23.2 ppm, with a sulfur removal rate as high as 88.4%. In addition, the catalyst could be directly recycled after vacuum drying, and the catalytic activity did not change significantly after five times of repeated use.

## 4. Summary and Outlook

The urgency of environmental and health concerns has driven the rapid development of alternative desulfurization technologies. In this paper, the methods of hydrodesulfurization, oxidative desulfurization, extractive desulfurization, extractive oxidation desulfurization, and catalytic oxidation desulfurization are reviewed, which provides optimized process conditions for future research.

Many factors affecting the desulfurization of ILs were determined, including the temperature, time, recovery of ILs, structural properties of sulfur, oxidizer, and molar ratio and mass ratio of the ILs and catalyst. The paper also studies the recovery and utilization of ILs. The results show that ILs can be used many times, which is the key to the rational use of the ILs desulfurization process. However, as the number of cycles increases, the efficiency decreases, mainly due to the increased oxidation products in the separation methods used, resulting in the loss of ILs. The relevant literature shows that the molar ratio of oxidizer-to-sulfur directly affects the desulfurization efficiency. In addition, the mass ratio of the ILs and catalyst has a limited influence on the desulfurization efficiency, and exceeding or failed to meet the optimal ratio may lead to reduced efficiency. A review of the literature on the effect of the catalyst mass and molar ratio on desulfurization efficiency shows that, in most cases, the greater the amount of the catalyst, the lower the desulfurization efficiency, because the excess catalyst cannot be effectively dissolved, resulting in a decrease in efficiency.

Although the ILs cleaning process and engineering research has proved the feasibility of the technology, there are still many key technical problems to be solved in the application of ILs in the industrialization process, such as the study and evaluation of ILs in the environment, safety, health, and long-term industrial operation stability, and how to efficiently separate the product from the ILs and achieve the recycling of the ILs. However, the study of ILs shows that it is not volatile, has the dual function of both solvent and catalyst, and can be used repeatedly, which highlights its excellent performance in the field of fuel desulfurization and its broad application prospects.

## Data Availability

Not applicable.
